# Brain Injury Associated Shock: An Under-Recognized and Challenging Prehospital Phenomenon

**DOI:** 10.1017/S1049023X24000359

**Published:** 2024-06

**Authors:** Christopher Partyka, Alexander Alexiou, John Williams, Jimmy Bliss, Matthew Miller, Ian Ferguson

**Affiliations:** 1. Staff Specialist in Prehospital & Retrieval Medicine, NSW Ambulance, Aeromedical Operations, Bankstown Aerodrome, NSW, Australia; 2. Staff Specialist in Emergency Medicine, Royal North Shore Hospital, St Leonards, NSW, Australia; 3. Clinical Lecturer and PhD Candidate, Faculty of Medicine and Health, University of Sydney, Sydney, Australia; 4. Consultant in Emergency Medicine, Royal London Hospital, London, England; 5. Consultant, Physician Response Unit, London’s Air Ambulance, London, England; 6. Emeritus Prehospital Doctor, Essex & Herts Air Ambulance, England; 7. Critical Care Paramedic, NSW Ambulance, Aeromedical Operations, Bankstown Aerodrome, NSW, Australia; 8. Staff Specialist in Emergency Medicine, Liverpool Hospital, Liverpool, NSW, Australia; 9. Conjoint Lecturer, St George and Sutherland Clinical Campus, University of New South Wales, NSW, Australia; 10. Anesthetist, St George Hospital, Sydney, Australia; 11. Conjoint Senior Lecturer, South West Sydney Clinical School, University of New South Wales, NSW, Australia

**Keywords:** brain injury associated shock, Emergency Medical Services, shock, trauma, traumatic brain injury

## Abstract

**Objective:**

Hemodynamic collapse in multi-trauma patients with severe traumatic brain injury (TBI) poses both a diagnostic and therapeutic challenge for prehospital clinicians. Brain injury associated shock (BIAS), likely resulting from catecholamine storm, can cause both ventricular dysfunction and vasoplegia but may present clinically in a manner similar to hemorrhagic shock. Despite different treatment strategies, few studies exist describing this phenomenon in the early post-injury phase. This retrospective observational study aimed to describe the frequency of shock in isolated TBI in prehospital trauma patients and to compare their clinical characteristics to those patients with hemorrhagic shock and TBI without shock.

**Methods:**

All prehospital trauma patients intubated by prehospital medical teams from New South Wales Ambulance Aeromedical Operations (NSWA-AO) with an initial Glasgow Coma Scale (GCS) of 12 or less were investigated. Shock was defined as a pre-intubation systolic blood pressure under 90mmHg and the administration of blood products or vasopressors. Injuries were classified from in-hospital computed tomography (CT) reports. From this, three study groups were derived: BIAS, hemorrhagic shock, and isolated TBI without shock. Descriptive statistics were then produced for clinical and treatment variables.

**Results:**

Of 1,292 intubated patients, 423 had an initial GCS of 12 or less, 24 patients (5.7% of the original cohort) had shock with an isolated TBI, and 39 patients had hemorrhagic shock. The hemodynamic parameters were similar amongst these groups, including values of tachycardia, hypotension, and elevated shock index. Prehospital clinical interventions including blood transfusion and total fluids administered were also similar, suggesting they were indistinguishable to prehospital clinicians.

**Conclusions:**

Hemodynamic compromise in the setting of isolated severe TBI is a rare clinical entity. Current prehospital physiological data available to clinicians do not allow for easy delineation between these patients from those with hemorrhagic shock.

## Introduction

Traumatic brain injury (TBI) is the leading cause of death and disability following trauma, which also delivers a significant economic and welfare burden.^1^ Isolated severe TBI can result in significant hemodynamic compromise. This brain injury associated shock (BIAS) may occur in 13% of adult trauma populations^2^ and 40% of major pediatric trauma.^3^

The exact mechanism by which BIAS is triggered remains unclear, but it is thought to be a brain-heart interaction involving structures including cortex, insula, hippocampus, and brain stem.^4^ The resultant catecholamine storm and sympathetic overload can lead to either systemic vasoconstriction or vasoplegia as well as increases in cardiac afterload, myocardial workload, and oxygen demand.^5,6^ The ensuing hypotension serves to magnify the catastrophic neurological injury.^7^ This clinical entity is distinctly different to bradycardic hypotension of neurogenic shock seen in acute spinal cord injury resulting from a loss of sympathetic vasomotor tone.

Trauma patients with brain injury and potential BIAS remain a diagnostic and therapeutic challenge for prehospital clinicians. While the optimization of cerebral blood flow remains the mainstay of treatment to prevent secondary brain injury, currently these patients receive empiric resuscitative therapies mostly targeting a hemorrhagic cause, including volume replacement with blood transfusion and osmotherapy (such as hypertonic saline) in addition to standard interventions such as rapid sequence intubation (RSI) and pleural decompression. The ability to recognize BIAS would be helpful to optimize the neuroresuscitation of these patients whilst rationalizing or withholding other potentially harmful empiric interventions. In a retrospective prehospital case series of isolated TBI patients only, 44% were cardiovascularly unstable, of which eight percent received a blood transfusion;^8^ however, no comparison to bleeding trauma patients was provided. It remains unclear how or if patients with BIAS are clinically distinguishable from those with hemorrhagic shock.

This study examines a population of prehospital trauma patients with hemodynamic compromise and isolated TBI and seeks to identify differences in their clinical characteristics compared to patients with hemorrhagic shock.

## Methods

### Study Design and Setting

A database review was conducted of all prehospital trauma patients intubated by New South Wales Ambulance Aeromedical Operations (NSWA-AO; New South Wales [NSW], Australia) from January 1, 2016 through December 31, 2019.

As a physician-paramedic prehospital and retrieval medicine service, NSWA-AO treats approximately 1,200 injured patients annually across NSW utilizing road ambulances plus rotary and fixed-wing aircraft. Physicians are specialists or final-year doctors-in-training, and all undergo intensive training in service models at the commencement of employment. Paramedics are permanent staff members who undertake the same induction training as physicians. All practitioners adhere to standard operating procedures and undergo regular currency training. All patient encounters are recorded in a clinical database (AirMaestro; Avinet, Australia) with paper-based case sheets and patient observations also uploaded to this database.

This study was approved by the South Western Sydney Local Health District Human Research Ethics Committee (Liverpool BC, NSW; 2022/ETH00212) and a waiver of consent was provided.

### Selection of Participants

This study is a pre-planned subgroup investigation of the Greater Sydney Area Helicopter Emergency Medical Services (GSA-HEMS; Bankstown Aerodrome, NSW) airway database,^9^ a retrospective observational study which included all NSWA-AO patients who underwent attempted tracheal intubation with ketamine as the induction agent in the years from 2016 through 2019. Clinical data were downloaded from AirMaestro and uploaded to a study-specific database where they were matched to detailed physiological vital sign recordings transformed from the operational Zoll X-series monitors (Zoll Medical Corporation; Tokyo, Japan) as well as relevant imaging results (prehospital point-of-care ultrasound and in-hospital computed tomography [CT] scans). Inter-rater reliability for this data extraction was assessed by cross-checking a random 10% of completed records with a second investigator using Cohens Kappa. These results were strong (0.8–0.9) or near-perfect (0.9) in all domains. The complete data extraction protocol and reliability analysis for these data are available elsewhere.^10^ Patients were included from this database who had an initial Glasgow Coma Scale (GCS) of 12 or less, suggestive of a moderate-to-severe TBI. Patients who were intubated with a sedative other than ketamine and those who underwent intubation without induction drugs during traumatic cardiac arrest were not included in the original airway database and therefore were also excluded from this cohort.

### Definitions

The following definitions were used. Prehospital shock was defined as a systolic blood pressure less than 90mmHg, requiring administration of either blood products (packed red cells or plasma) or inotropic agents (epinephrine, norepinephrine, or metaraminol).

An extracranial life-threatening injury which could be responsible for hemorrhagic, hypovolemic, or spinal-mediated neurogenic shock was based on CT imaging on arrival to hospital. These included:

- Hemorrhage
  - Major thoracoabdominal bleeding (hemothorax, hemoperitoneum, or solid organ injury requiring interventional hemorrhage control);
  - Extensive orthopedic injuries (more than one long bone fracture or unstable pelvic fracture with documented hematoma or requiring interventional hemorrhage control); and/or
  - Documented external hemorrhage on scene.
- Neurogenic shock (acute spinal injury with motor deficit).
- Obstructive shock (tension pneumothorax or cardiac tamponade).
- No obvious pre-existing condition causing shock (anaphylaxis and sepsis).

Intracranial pressure (ICP) was considered to be elevated when the radiology report listed herniation (subfalcine, transtentorial, uncal, or cerebellar [tonsillar]), midline shift, or significant mass effect with either subdural hematoma, epidural hematoma, cerebral contusion, or traumatic subarachnoid hemorrhage.^11^

### Study Groups

Patients in this cohort were allocated to three pre-defined groups. The *BIAS* group included shocked patients with TBI described on CT and no evidence of concurrent extracranial life-threatening injury. The *Hem-Shock* group included shocked patients with evidence of hemorrhage, whilst the *NonShock-TBI* group included patients with TBI and no evidence of shock.

### Outcomes

The primary outcome was to describe the frequency of shock in patients with a moderate to severe TBI (GCS of ≤12) and no extracranial cause of shock. Secondary outcomes of this study included: to describe the demographics, physiological characteristics, and clinical management of patients with *BIAS* and to compare them against both *Hem-Shock* and *NonShock-TBI* groups; as well as to sub-categorize the *BIAS* group and compare those who did and did not have features of raised ICP on CT imaging.

### Analysis

Statistical analyses were conducted using R Environment for Statistical Computing (v4.2.1; R Core Team 2022; Vienna, Austria). Categorical data were reported as frequency (n) with percentages (%), with continuous data reported as medians with interquartile range (IQR). The chi-squared test was used to compare between-group categorical variables, whereas the Student’s t-test or the Mann-Whitney test were used to compare differences in continual variables, dependent on the normality of distribution. Statistical significance was set with an alpha of 0.05.

## Results

**Figure 1.**
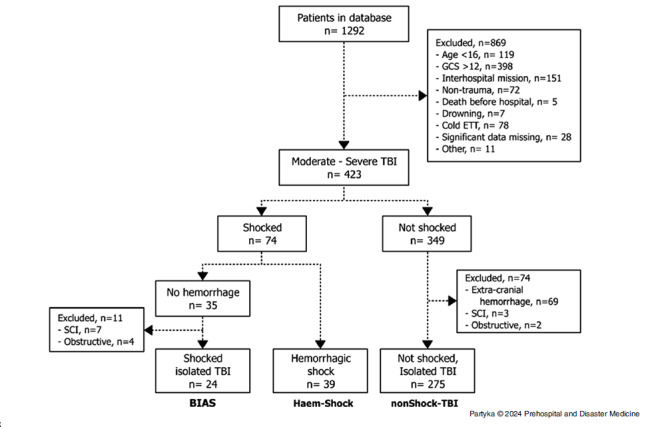
Consort Diagram. Abbreviations: GCS, Glasgow Coma Scale; ETT, endotracheal tube; TBI, traumatic brain injury; SCI, spinal cord injury.

Over the four-year study period, a total 1,292 patients underwent RSI by NSWA-AO medical teams, of which there were 423 prehospital trauma patients with an initial GCS of 12 or less (Figure 1). Of these, 74 (17.5%) were classified as shocked. The *BIAS* group comprised 24 patients (5.7%), the *Hem-Shock* group 39 patients (9.2%), and 275 patients were categorized into the *NonShock-TBI* group (65%). The remainder were not shocked, non-TBI, and were excluded from further analysis.

The demographics and clinical characteristics of patients in the three study groups are shown in Table 1. Most patients were male, and the median age was 36-41 years. The predominant mechanism of injury was road traffic accidents. The hemodynamic parameters in the *BIAS* and *Hem-Shock* groups were indistinguishable, including tachycardia, systolic and diastolic hypotension, and elevated shock index, as was the frequency of anisocoria. Prehospital clinical interventions were also similar between these groups, including the use of blood transfusion in all but one patient. The overall volume of fluids received were again similar between *BIAS* and *Hem-Shock* groups, including both total number of blood product units transfused and total volume of intravenous crystalloid administered. The *NonShock-TBI* group demonstrated near normal hemodynamics, required far fewer clinical interventions (including transfusion), and received more substantial induction doses to facilitate intubation.

**Table 1.** Demographics and Clinical Characteristics

Characteristic	BIAS N = 24	Hem-Shock N = 39	NonShock-TBI N = 275
Age (years)	41 (IQR = 23, 63)	37 (IQR = 26-57)	36 (IQR = 25-54)
Gender			
Female	5 (20.8%)	11 (28.2%)	60 (21.8%)
Male	19 (79.2%)	28 (71.8%)	215 (78.2%)
Weight (kg)	80 (IQR = 70-82.5)	75 (IQR = 70-80)	80 (IQR = 70-90)
Mission Category			
Road Traffic Accident	16 (66.7%)	25 (67.6%)	139 (50.9%)
Fall	3 (12.5%)	3 (8.1%)	28 (10.3%)
Pedestrian	2 (8.3%)	3 (8.1%)	14 (5.1%)
Industrial	1 (4.2%)	3 (8.1%)	16 (5.9%)
Other	2 (8.3%)	3 (8.1%)	76 (27.6%)
Arrival Vital Signs			
GCS	6 (IQR = 3-8)	6 (4, 8)	7 (4, 9)
GCS (motor)	2 (IQR = 1-4)	3 (1, 4)	4 (2, 5)
Pulse Rate	120 (IQR = 94-139)	125 (IQR = 106-140)	99 (IQR = 80-112)
Systolic BP (mmHg)	70 (IQR = 49-80)	71 (IQR = 60-81)	132 (IQR = 116-150)
Diastolic BP (mmHg)	40 (IQR = 30-47)	49 (IQR = 40-54)	80 (IQR = 70-88)
Pulse Oximetry (%)	90 (IQR = 79-98)	94 (IQR = 82-98)	98 (IQR = 95-100)
Respiratory Rate	21 (IQR = 18-25)	20 (IQR = 17-30)	18 (IQR = 14-22)
Shock Index	1.8 (IQR = 1.4-2.2)	1.8 (IQR = 1.3-2.2)	0.7 (IQR = 0.6-0.9)
Pulse Pressure (mmHg)	25 (IQR = 16-35)	23 (IQR = 19-35)	52 (IQR = 40-70)
Pupils			
Anisocoria^a^ (arrival)	9.0 (37.5%)	13.0 (33.3%)	48.0 (17.5%)
Anisocoria^a^ (at destination)	10.0 (41.7%)	9.0 (23.1%)	52.0 (18.9%)
Prehospital Treatment			
Blood Transfusion	24 (100.0%)	38 (97.4%)	33 (12.0%)
TXA	18.0 (75.0%)	34.0 (87.2%)	30.0 (10.9%)
Thoracostomy	7.0 (29.2%)	18.0 (46.2%)	28.0 (10.2%)
Tourniquet	2.0 (8.3%)	3.0 (7.7%)	2.0 (0.7%)
Osmotherapy	9 (37.5%)	14 (35.9%)	54 (19.6%)
Prehospital eFAST			
Peritoneal FF	2 (8.3%)	10 (25.6%)	10 (3.6%)
Pneumothorax	1 (4.2%)	8 (20.5%)	18 (6.5%)
Fluid Therapy			
RBC (pre-RSI) (units)	1 (IQR = 0-2)	2 (IQR = 1-2)	0 (IQR = 0-0)
RBC (total) (units)	3 (IQR = 3-3)	3 (IQR = 3-3)	0 (IQR = 0-0)
FFP (total) (units)	0 (IQR = 0-0)	0 (IQR = 0-2)	0 (IQR = 0-0)
Crystalloid (pre-RSI) (mL)	375 (IQR = 212.5-500)	750 (IQR = 500-1000)	250 (IQR = 0-500)
Crystalloid (post-RSI) (mL)	250 (IQR = 50-500)	500 (IQR = 250-687.5)	400 (IQR = 200-500)
Induction Agents			
Ketamine (mg)	50 (IQR = 40-100)	50 (IQR = 40-80)	100 (80, 130)
Fentanyl (mcg)	20 (IQR = 0-50)	50 (IQR = 0-100)	50 (0, 100)
Rocuronium (mg)	150 (IQR = 120-150)	150 (IQR = 102.5-160)	150 (IQR = 120-150)
Inotropes Administered	3 (12.5%)	5 (12.8%)	6 (2.2%)

Note: Median (IQR); n/N (%).

Abbreviations: BIAS, Brain Injury Associated Shock; Hem-Shock, hemorrhagic shock; NonShock-TBI, traumatic brain injury without shock; GCS, Glasgow Coma Score; BP, blood pressure; TXA, tranexamic acid; FF, free fluid; RBC, red blood cells; RSI, rapid sequence intubation; FFP, fresh frozen plasma; ICP, intracranial pressure.

a Anisocoria: non-reactive pupil ≥2mm larger than reactive pupil, or both pupils non-reactive.

Of the 21 patients in the *BIAS* group with available CT reports, six (25%) were identified as having features of raised ICP on CT imaging, whereas 15 (75%) did not. Their clinical characteristics are presented in Table 2. The patients with features of raised ICP appeared to have a lower initial GCS combined with a more profound hemodynamic disturbance, including lower systolic blood pressures and a more exaggerated shock index (2.6 compared to 1.6 in the group without features of raised ICP).

**Table 2.** Clinical Characteristics of Patients with BIAS Delineated by Features of Raised ICP on In-Patient CT Imaging

Characteristic	No Raised ICP on CT N = 15	Raised ICP on CT N = 6
**Age (years)**	42 (IQR = 23.5-64)	30 (IQR = 23-60.2)
**Gender**		
Female	3 (20.0%)	2 (33.3%)
Male	12 (80.0%)	4 (66.7%)
**Weight (kg)**	80 (IQR = 72.5-95)	72.5 (IQR = 70-75)
**Mission Category**		
Road Traffic Accident	10 (66.7%)	4 (66.7%)
Fall	2 (13.3%)	1 (16.7%)
Pedestrian	2 (13.3%)	–
Industrial	1 (6.7%)	–
Shooting	–	1 (16.7%)
**Arrival Vital Signs**		
GCS	7 (IQR = 4-11)	4 (IQR = 3-6)
GCS (motor)	4 (IQR = 1-5)	2 (IQR = 1-3)
Pulse Rate	120 (IQR = 108-130)	146 (IQR = 133-153)
Systolic BP (mmHg)	76 (IQR = 67-83)	48 (IQR = 45-64)
Diastolic BP (mmHg)	42 (IQR = 39-54)	31 (IQR = 20-37)
Pulse Oximetry (%)	96 (IQR = 90-99)	82 (IQR = 65-86)
Respiratory Rate	22 (IQR = 17-25)	25 (IQR = 20-31)
Shock Index	1.6 (IQR = 1.4-1.9)	2.6 (IQR = 2-4.1)
Pulse Pressure (mmHg)	25 (IQR = 18-36)	25 (IQR = 15-31)
**Pupils**		
Anisocoria^3^ (arrival)	3 (20.0%)	3 (50.0%)
Anisocoria^3^ (at destination)	3 (20.0%)	4 (67.0%)
**Prehospital Treatment**		
Blood Transfusion	15 (100.0%)	6 (100.0%)
TXA	13 (86.7%)	3 (50.0%)
Thoracostomy	5 (33.3%)	1 (16.7%)
Torniquet	2 (13.3%)	–
Osmotherapy	3 (20.0%)	4 (66.7%)
Inotropes	1 (6.7%)	2 (33.3%)
**Induction Agents**		
Ketamine (mg)	65 (IQR = 40-100)	50 (IQR = 27.5-87.5)
Fentanyl (mcg)	100 (IQR = 100-100)	0 (IQR = 0-25)
Rocuronium (mg)	150 (IQR = 120-165)	150 (IQR = 120-150)

Abbreviations: BIAS, brain injury associated shock; GCS, Glasgow Coma Score; BP, blood pressure; ICP, intracranial pressure; CT, computed tomography; TXA, tranexamic acid.

## Discussion

In this case series of trauma patients requiring prehospital RSI with an initial GCS of 12 or less, 5.7% were identified as having BIAS. This demonstrates the infrequency with which BIAS occurs in practice, and given the physiological similarities between patients with BIAS and those with hemorrhagic shock, it is a challenge for prehospital clinicians to distinguish between these entities. Prehospital clinicians are more likely to observe multifactorial shock with both severe TBI and acute hemorrhage, warranting a combination of targeted treatments, and this may have led to the similarities in treatment given to both groups.

The incidence of BIAS in this cohort is lower than that previously reported in the literature and highlights the inherent difficulty in studying these challenging patients. This may be due to the inclusion criteria used to define these cases or the clinical case mix treated in this cohort. A previous prehospital study^8^ included only patients with clinical signs of TBI and CT-proven TBI, although the clinical signs were not defined. A broader criterion was chosen here of GCS of 12 or less undergoing a prehospital RSI; however, the TBI may not have been the main indication for intubation, resulting in a larger denominator. Shock was also defined as hypotension combined with administration of either vasopressors or blood product transfusion. While this resulted in nearly all shocked patients receiving blood (TBI or not), it indicates that vasopressors alone were not considered in patients who were shocked with an isolated TBI. Whether this was due to difficulties establishing an isolated TBI as the diagnosis, or clinicians not comfortable withholding blood products in unstable trauma patients, is unclear.

Point-of-care ultrasound is an imaging modality available to prehospital clinicians that may assist in visualizing the underlying mechanism of shock by delineating profound hypoperfusion with compensative vasoconstriction against those with severe vasoplegia. Ultrasound can also identify compartmental hemorrhage such as hemoperitoneum and hemothorax. Echocardiographic assessment of these patients has revealed an array of findings including ventricular suppression, hyperdynamic response to vasoplegia, and left ventricular outflow tract obstruction. Evidence suggests that once at hospital, the utilization of echocardiography in these patients is low; however, the information derived from its use is associated with lower in-hospital mortality.^12^ Prehospital echocardiography may be the key to optimizing the hemodynamics of these complex cases.

## Limitations

This study has several limitations which are important to discuss. Firstly, the cohort in this study is very small, which is representative of an uncommon disease process. This limits identification of subtle differences and patterns of physiological derangement. Further prospective study of this pathology with a larger sample size is needed if meaningful differences are going to be identified, and multi-organization collaboration may be needed.

Secondly, patients who underwent intubation without induction drugs were not included in this dataset. In this service, cold intubation is only performed on patients in cardiac arrest. This likely resulted in some missed cases, as it is possible that some individuals were successfully resuscitated and survived to hospital with severe TBI.

Finally, these data do not account for patients with concurrent hemorrhage and brain injury whereby dual pathology may be driving the shocked state. Future study is required in this cohort to help identify the predominant cause of hemodynamic instability in such individuals to assist in customizing their resuscitation.

## Conclusions

Hemodynamic compromise due to isolated severe TBI is a rare but potentially important clinical condition. Prehospital clinical data available do not allow for the rapid delineation between these patients from those with hemorrhagic shock. Further prospective data are required on a larger scale to help separate these groups in order to guide more accurately targeted therapies.
